# The legacy of Dr Marjory Warren's publications

**DOI:** 10.1177/09677720241273643

**Published:** 2024-08-28

**Authors:** David B. Hogan

**Affiliations:** Departments of Medicine and Community Health Sciences, Cumming School of Medicine, University of Calgary, HSC-3330 Hospital Dr NW, Calgary, Alberta, Canada

**Keywords:** Geriatric medicine, bibliography, aged, delivery of healthcare

## Abstract

While the contributions of Dr Marjory W. Warren to geriatric medicine are widely acknowledged, their specifics have become obscured by the passage of time. The primary objective of this narrative review of her medical publications was to clarify the contributions she made for this field of medical practice. A total of 82 publications were found. In them Warren presented a then novel and hopeful approach to the management of older patients that included making care plans derived from comprehensive assessments, implementing team-based interventions, and ensuring continuity of care. These innovations, though, took years to implement and included what would now be considered a number of paternalistic and hierarchical aspects. Objective patient outcome data was rarely presented. While responsible for innovations that remain key to the field, some of what she proposed are either no longer possible (e.g. large in-patient units with prolonged lengths of stay) or have required modifications to align with current practice.

## Introduction

There is no question about the historical importance of Dr Marjory W. Warren (28 October 1897–5 September 1960) to geriatric medicine.^1–4^ She was trained in medicine at the London (Royal Free Hospital) School of Medicine for Women and passed the Conjoint Board examinations (qualifying with a basic L.R.C.P. Lond, M.R.C.S. Eng) in 1923. She received no higher degree and was described by a colleague as a “sound practical doctor.”^1^ After a number of junior medical posts, she joined the West Middlesex Hospital (WMH), a municipal hospital, as an assistant medical officer in 1926. Five years later she was promoted to deputy medical director and became a consultant physician in 1949. Her clinical interests at the WMH were initially surgery before shifting to medicine and finally geriatrics.^1^ In 1935 an adjacent workhouse was taken over by the WMH. Medical responsibility for the care of the mostly older residents was assigned to Warren who was then in her late 30s. Taking a fresh approach to the gloomy conditions encountered, Warren began her pioneering work in geriatrics that will be summarized later. Before the steps taken by Warren and a few contemporaries, the care of older patients with chronic health issues in Britain was primarily custodial.^3^ The then new approach she advocated for showed that with appropriate care a sizable proportion could be discharged back to the community. Key components of this remain relevant to current geriatric practice.^4^

A charismatic, strong-willed, and tenacious person, Warren favoured action over detailed study, which fitted the needs of the National Health Service (NHS) as it was launched in 1948 when there were concerns that it might be swamped by older patients. With the publication of her initial papers on geriatrics and by word-of-mouth, the fame of Warren and her service spread rapidly, attracting many visitors to the WMH. For her services, she was appointed Commander of the Order of the British Empire (C.B.E.) in 1959. The next year Warren was fatally injured in a motor vehicle crash while on her way to a medical meeting at Baden-Baden in Germany.

With the passage of time, though, the specifics of her contributions have become vague and almost mythical in nature. Unless already obtained, the testimony of her contemporaries has slipped beyond our reach, but her medical publications do provide a window on the past. The extent of this record is unclear. Dale A. Matthews stated her “entire corpus of published works” amounted to 28,^2^ but both George F. Adams and M.J. Dunham described it as extensive.^1,3^

The primary objective of this narrative review of her medical publications was to develop a better understanding of how she cared for older patients. A secondary one was to reflect on how much of her approach has been retained, modified or dropped.

## Methods

The publications listed by Matthews^2^ were examined first. PubMed and Google Scholar database searches using variants of Warren's name (e.g. Warren MW, Marjory Warren) as search terms were then conducted for other publications appearing between 1 January 1926 (the year she began working at the WMH) and 31 December 1961 (to capture items in press at the time of her death). Geriatric textbooks, journal indices (*British Medical Journal*, *Geriatrics*, *Journal of Gerontology*, *The Lancet*), and geriatric/ gerontology bibliographies^5–7^ were next searched. Citation mining of selected manuscripts was conducted. Correspondence was exchanged with two of the schools she attended (North London Collegiate School, London [Royal Free Hospital] School of Medicine for Women) and Dr Ronald Bayne,^8^ then her last known surviving house officer, requesting assistance. Publications that acknowledged Warren's assistance to their writing or underlying research, included transcriptions of her comments, or provided detailed information of one of her presentations were retrieved for review but not included as an authored publication.

The sole inclusion criterion was whether Warren authored or co-authored the publication. Attempts were made to obtain full text copies of all identified English-language publications to verify authorship and review their contents. Contributions were categorized as to publication date (1926–1950, 1951–1955, 1956–61), authorship (single, multiple), source (journal, other), and primary topic (geriatric services, geriatric rehabilitation, community care/housing, clinical aspects [i.e. approach to assessment/management of diseases or syndromes/conditions], book or literature reviews). Though limited by small sample sizes, exploratory chi-square testing of categorical data was done without correction for multiple comparisons.

## Results

Two of the publications listed by Matthews^2^ were excluded after review. The total identified through all means was 82 publications. As shown in Table i (Supplemental material) they were predominantly single-authored (89% of the total) journal articles (73%). Her work appeared in 30 different journals (the *British Medical Journal* with 12 publications was the most favoured venue). While the primary audience for her introductory papers on geriatric services was initially domestic, this became more international (i.e. European nations, the United States, Canada, Australia) later on. Finally, the proportion of her publications categorized as dealing with clinical aspects increased as the years passed (p = 0.028). The clinical condition Warren wrote most about was cerebrovascular disease/hemiplegia (n = 7).

During the early years of the NHS Warren with others advocated for an integrated system of care that incorporated acute hospital wards reserved for older patients (with admissions controlled by the geriatric service), long-stay annexes (for the “residuum” of patients who survived but could not be discharged), close liaison with residential facilities, and community out-reach services (i.e. clinics, domiciliary visits).^9^ This was never implemented as originally planned. Warren's focus was on in-patient care (planning for an out-patient arm to her services only began in 1948). Innovations she developed included a classification system that permitted grouping like patients on wards staffed and equipped for their needs, upgrades to the wards, individual care plans based on comprehensive (i.e. medical, psychological, social) assessments, physician-led team-based interventions that included prevention and active rehabilitation utilizing simple techniques (e.g. bed-end exercises^10^) and appliances (a number of which she developed^11^), and an emphasis on continuity of care. She railed against “defeatist” attitudes and indiscriminate use of bed rest. Warren insisted that the care of older patients required an optimistic, hopeful attitude coupled with meticulous attention to detail.

While in 1943 she supported recognizing geriatrics as a specialty (she wrote that unless this occurred geriatrics “will not receive the sympathy and attention it deserves”^12^), by the end of her career she seemingly lost this conviction (in 1960 she stated geriatrics ‘must remain an integral part of general medicine if it is to fulfill its proper function'^13^). Warren placed demands on her patients. She felt no older patient should have anything done for them that they could do for themselves. To enforce this, she was willing to “irritate” them if needed. When asked in a panel discussion whether she would use “therapeutic rage as a stimulant” for participation in rehabilitation, she responded yes.^14^ Likewise it was also clear who was in charge of the therapeutic team. The physician had ultimate responsibility for the patient's care plan. If there was a difference of opinion between staff and the physician, the physician “should explain carefully the underlying rationale and why such a regime is best and this must be then followed.”^15^

Material was often reused in her publications. A minimum of 14 were first given as oral presentations before appearing in print, and there was substantial overlap in both the themes and text found in her writings. Numerical data on overall patient outcomes for the WMH geriatric unit was only provided once.^16^ Between 1944 and 1946 (inclusive) two of her wards (one female, one male) admitted 1364 patients. Five hundred and eighty-nine (43%) died in hospital, 349 (26%) were discharged home, 137 (10%) went to a residential facility, and 289 (21%) remained as a “residuum.”^16^ No additional patient characteristics or comparison data were provided. The unstated assumption was very few if any would have been discharged home or to a residential facility if not for the care provided. Though these results date from a self-admitted atypical time (the end of the Second World War),^16^ updated figures were never provided.

Warren was acknowledged, extensively quoted, or had one of her presentations comprehensively described in a further 14 publications.^17–30^ Five containing additional data on her practice are summarized in the following paragraphs.

Diathermy (controlled production of deep heating by electrical currents) was utilized for the treatment of pneumonia during the last century. In a 1929 review of treatment results, C.A. Robinson reported the 89 cases of lobar pneumonia treated with diathermy at the WMH ‘have been chiefly under the immediate charge of Dr Marjory Warren, and it is owing to her zeal that the notes of the cases have been so complete.'^17^ Harry E. Stewart quoted Warren as saying the WMH had set up a diathermy ward for pneumonia, which was used at full capacity to treat 50+ Welsh hunger marchers.^18^ Through exposure and undernutrition they had a poor prognosis, but Warren reported a low mortality rate of 3%. Stewart and Warren likely discussed this when they met on 3 December, 1934 during her first American visit.^31^

At a 1950 talk given at the Hope Hospital (Salford, England) Warren made a ‘plea for the retention of freedom of method' for pioneering geriatric units.^24^ Other than age, distinguishing features of patients were the multiplicity of conditions (with ‘the treatment of one condition often conflicting with another’), long length of stays, and ‘insecurity of the future' (i.e. uncertainty whether they would return home). Early social work involvement was recommended while physical interventions had to be both relevant and adapted for the patient. Admission to geriatric units should only be after consultation, as ‘the old happy days of “dumping” had gone.' The extended treatment team would ‘perhaps [include] a hairdresser for the women and a barber for the boys' while its “skipper” should be a physician responsible for treating the patient, coordinating the team, and integrating the unit within the host hospital. Only by ‘treating large numbers … one could perfect one's methods and learn the prognosis.' Warren advised against the rapid establishment of geriatric units. With few qualified physicians, the appointment of ‘labelled geriatricians … [who] neither knew what to do or what could be done' would be disastrous.

In the transactions of the 1951 Macy Conference *Problems of Aging*,^25^ Warren commented on the goals of gerontology, presence of a “defeatist” attitude towards the care of older persons, need for more research on “irremediable” cases, appropriateness of financial assistance for older persons living in ‘expensive living quarters,' and use of 65 as a compulsory retirement age. Warren felt geriatric units in England through ‘active rehabilitation and … a much sharper medical focus' had led to a reduced need for beds, but the degree of success for individual units ‘depends upon your inheritance … [if] you were handed two hundred patients who were truly bedridden, obviously the turnover of these patients would be nearly nil except by death … [if] less bedridden, your turnover would be better.' As for the duration of improvements, hemiplegic patients maintained functional gains if another stroke didn’t occur, and out-patient supervision was provided. She noted the importance of having an out-patient arm ‘partly run for assessment and partly for follow-up.'

Milton and Margaret Silverman interviewed Warren shortly before her death.^30^ Prior to the WMH assuming control of Warkworth House in 1935, hospital physicians referred to it as *The Other Side*. The only service provided was a doctor visiting every few days to ask if anyone was acutely ill. Warren was tasked with determining what should be done. Not knowing the residents, she examined over 500 of them. While not doing a ‘complete job' on everyone, at the end of six months she had a ‘reasonably good picture.' Over the subsequent eight months she laboriously developed a scheme of grouping residents according to medical and nursing needs that the Medical Superintendent grudgingly agreed with. Warren reassured him that if it ‘doesn’t work out, I’ll carry the patients back' to their original wards. She started with those who had strokes, as most then felt they could not be helped. Some showed encouraging improvements with rehabilitation. Within a few years attitudes and results improved. Guaranteed readmissions, vacation stays, and out-patient services were implemented. Local general practitioners came to trust the service while London physicians brought their parents to it. Active treatment was the key – ‘Unless you get these old patients up and out, they’ll silt up any hospital and bankrupt any medical programme in the world.' The Silvermans felt the ‘excitement in its old wards and narrow corridors' on a visit. While some patients came to die, ‘the overwhelming majority had come to be cured.' Warren and the staff on the geriatric unit showed that a ‘patient is not beyond hope merely because he's beyond sixty-five.'

## Conclusion

In her writings Warren made a strong case for a new approach (what she referred to as “radical reform”^32^) to the care of older patients on the basis of equity, effectiveness, and necessity. The presence of multiple morbidities and the importance of the patient's psychological state (i.e. presence of anxiety, depression, demoralization, conservation-withdrawal,^33^ and/or “insecurity of the future”) and existing social/ physical home environment were emphasized, as were the risks of bed rest. Treatment teams with positive attitudes under physician direction that provided medical interventions, rehabilitation, and discharge planning were the engine driving better care. To sustain these changes, a geriatric service had to control its admissions (which encountered opposition), maintain active patient flow through transferring those who could not be discharged home to long-stay units, and ensure continuity of care.

Initiating these changes with little to no guidance during the 1930s and the Second World War raised daunting challenges (such as being bombed)^34^ that she overcame. These circumstances, though, also provided opportunity, as it gave Warren the authority, access to space and resources (though limited), and uninterrupted time to develop her approach (she was thankful that ‘great events elsewhere had distracted attention from what she was doing' until her ‘ideas were marshalled and her system set in order'^1^). She also overcame the barriers raised by her gender (i.e. ‘she was a woman in a jungle of men') and the lack of membership in a Royal College.^35^

The pivotal role she envisioned for geriatric departments was overly ambitious, especially in light of the division of responsibility in England for medical and residential care, the small number of available geriatric units and staff (including physicians) for the task as described, and the caution expressed by Warren and others against rapid growth. The size and average length of stay (1–2 months^36^) of geriatric units in her era are not possible in ours. It is also important to note that while better care contributed, the “substitution of 200 beds with a turnover for 700 beds with stagnation”^22^ took years to fully accomplish^16,37^ and arose primarily from controlling admissions (i.e. being selective on who to take and not automatically filling beds after patients died, were transferred, or discharged).^16,37^

When assessing Warren and her accomplishments uncritical *presentism* (i.e. the introduction of present-day ideas and perspectives into depictions or interpretations of the past) must be guarded against. Our better understanding of diseases coupled with an expanded range of investigative and therapeutic options should be kept in mind when considering her high in-patient mortality rate^16^ and advice on clinical issues like, as an example, hypertension. She wrote it was ‘generally unwise, and often unkind to reveal the presence of hypertension to the patient, and often it is better not to discuss it even with relatives.'^38^ This was in line with then current clinical practice, which recommended that treatment with the limited armamentarium then available be limited to those with clinical evidence of cardiac difficulties.^39^ There also has been an evolution in our thinking about both physician–patient relationships (from paternalism to one hopefully more balanced)^40,41^ and the “command and control” management style of the early years of the National Health Service^41,42^ that could explain at least partially her attitudes towards patients and staff described earlier. Though we might disagree with them, there can be no questioning Warren's commitment to providing better medical care to older persons.

Equivalent changes have taken place in the standards of medical research and publishing. In 1982, nearly 40 years after Warren's initial description of a hospital-based geriatric service,^12^ Rubenstein et al. stated that ‘No country has yet performed a well-controlled study to evaluate the effectiveness of these [geriatric] units'^43^ and concluded that ‘more rigorous studies [e.g., randomized controlled trial] are needed [of geriatric units] … to better document these optimistic early reports.'^43^ In response, Powell noted that ‘British geriatricians who had the regrettable experience of caring for elderly people in hospital before the advent of geriatric services have no doubt of these units’ effectiveness.'^44^ What Warren provided as proof of the effectiveness was appropriate for her time and circumstances and should not be judged by current research standards. As for publications, in the 1940s and 1950s duplicate publications were not uncommon (e.g. the previously referenced paper on diathermy by Robinson^17^ was republished in another journal a year later^45^).

The gaps in her writings are also interesting. Notwithstanding a 40–45% death rate, Warren never wrote about end-of-life care other than recommending a stepped approach to drug selection for malignant pain.^46^ Concerns about defeatist attitudes and her belief that older people were almost duty-bound to be as independent as possible did not easily align with C.M. Saunders’ (1918–2005) comment that there comes a time ‘when active treatment of all kinds becomes increasingly irrelevant to his [the dying patient's] real needs.'^47^ The separation of geriatrics from end-of-life (or palliative) care during this time has been previously commented upon.^48^ She also wrote relatively little on the geriatric syndromes (i.e. frailty, confusion, falls, incontinence) that are current preoccupations of the field.^49^

Strengths of this article are the systematic approach taken to identifying her publications and the detailed review of them. While it is nearly certain there are additional non-identified publications, it is unlikely their inclusion would substantially change the findings presented. A limitation is having all reviews done by one person. This was somewhat counteracted by concurrently examining the highlights and interpretations of her career made by Warren's biographers.^2,3^

The written word provides a limited perspective of Dr Warren. The impact of her personality and inspiring example on others is missing. Dr Nanette H. Nisbet, in the introduction of her MD thesis, wrote that when the ‘metamorphosis [on Warren's unit] of an utterly hopeless helpless patient into an active energetic and exceedingly grateful one' was demonstrated to visiting physicians and nurses ‘it was not surprising that the spectators could never refrain from spontaneous outbursts of applause.'^50^

## Supplemental Material

sj-docx-1-jmb-10.1177_09677720241273643 - Supplemental material for The legacy of Dr Marjory Warren's publications Supplemental material, sj-docx-1-jmb-10.1177_09677720241273643 for The legacy of Dr Marjory Warren's publications by David B. Hogan in Journal of Medical Biography
