# Supplementary material for: The legacy of Dr Marjory Warren's publications
Source: J Med Biogr. 2024 Aug 28;33(3):209–14. doi: 10.1177/09677720241273643 (PMC12241693; doi:10.1177/09677720241273643)
Supplement: sj-docx-1-jmb-10.1177_09677720241273643 - Supplemental material for The legacy of Dr Marjory Warren's publications [file sj-docx-1-jmb-10.1177_09677720241273643.docx]

**SUPPLEMENTAL MATERIAL (August 28, 2024)**

**TABLE i -** Summary of select publication characteristics.

| **Year Range** | **Up to 1950 (inclusive)** | **1951-1955** | **1956 and up** | **Total** |
| --- | --- | --- | --- | --- |
| **Number of Publications** | 23 | 28 | 31 | 82 |
| **Number (%) Single Authorship** | 21 (91% of total) | 24 (86%) | 28 (90%) | 73 (89%) |
| **Number (%) of Journal Articles** | 19 (83% of total) | 21(75%) | 20 (65%) | 60 (73%) |
| **Publication Topic, Number** | - Geriatric Services 12 - Geriatric Rehab.^a^ 4 - Community Care/ Housing 1 - Clinical Aspects 1 - Book/ Literature Reviews 1 - Other 4 | - Geriatric Services 2 - Geriatric Rehab. 2 - Community Care/ Housing 5 - Clinical Aspects 6 - Book/ Literature Reviews 5 - Other 8 | - Geriatric Services 7 - Geriatric Rehab. 2 - Community Care/ Housing 1 - Clinical Aspects 11 - Book/ Literature Reviews 6 - Other 4 | - Geriatric Services 21 - Geriatric Rehab. 8 - Community Care/ Housing 7 - Clinical Aspects 18^b^ - Book/ Literature Reviews 12 - Other 16 |

^a^Rehab. – rehabilitation

^b^Change in proportion of publications dealing with clinical aspects, p = 0.028

**Annotated list of articles (n = 82) arranged chronologically (earliest to most recent) authored by Dr. Marjory W. Warren.**

- Warren MW. Suicidal Cut Throat: Recovery. Brit Med J. 1931, 1:894.
  - Case report describing the non-operative management of a 30-year old male cared for by Warren in the West Middlesex Hospital (WMH). He attempted suicide by cutting his throat with a razor “in a moment of financial anxiety.”
    - The Brentford Union workhouse was built in 1837-8 with an infirmary erected in 1881-2 to the north of the workhouse and a second infirmary block added on the west side in 1888-9. In 1883 a workhouse school, Percy House, was erected to the south-west. At the outbreak of the First World War the school functioned as an auxiliary military hospital. After the War, it was used to store military records until 1922. In 1895-1902, the original workhouse was mostly demolished and replaced by a new infirmary and workhouse. From 1920 the infirmary was known as the WMH and the workhouse as Warkworth House. In 1935, the two were merged to create the West Middlesex County Hospital. Two hundred Warkworth residents were transferred to Percy House, which had been converted to a residential home. (Anonymous. The Workhouse - Brentford, Middlesex. Accessed June 3^rd^, 2024 at <https://www.workhouses.org.uk/Brentford/>; Anonymous. Lost Hospitals of London - West Middlesex University Hospital. Accessed June 3^rd^, 2024 at <https://ezitis.myzen.co.uk/westmiddlesex.html>)
- Warren MW. Care of chronic sick - a case for treating chronic sick in blocks in a general hospital. Brit Med J. 1943, 2(4329):822-23.
  - This was originally given as a presentation to the London and Home Counties Branch of the Medical Superintendents’ Society. In it, Warren advocated for treating older chronic sick patients (younger chronic sick patients would be cared for separately as they were irritated by older persons and vice versa) on specialized wards in general hospitals. This would create a setting where medical and nursing education, care requiring hospital facilities, and research could be done. The number of older chronic sick patients was increasing “from the steady practice of preventive and industrial medicine” and could be “no longer ignored.” Warren noted there were more women than men in institutional settings (one of the few times sex/gender came up in her writings). She wrote geriatrics needed recognition as a medical discipline if it was to receive the “sympathy and attention it deserves.” A classification scheme for patients (i.e., the categories were up-patients, continent bed-ridden, incontinent, “quietly restless and confused” patients, and “senile dements” requiring segregation) was presented. Grouping based on this scheme allowed the requirements of each group to be addressed. In advice on staffing, the need for experienced nurses was made. The unit should not be used “by the matron as punishment wards for nurses.” She closed by mentioning that geriatrics had “received more attention in America … [with] much of the literature on the subject” coming from that country.
- Warren MW. The Care of Old Sick People. Nursing Mirror. 1944, 80:47-48.
  - An abridged version of the preceding paper.
- Warren MW. Order of St. Olav. Isleworth League of Nurses’ Journal. 1945. In: *West Middlesex University Hospital – A History* (M. Black). The West Middlesex University Hospital N.H.S. Trust (Hounslow, Middlesex), 1993, page 53.
  - In 1940 reciprocal events began being held between WMH nurses and Norwegian merchant seamen. A number of marriages ensued. King Haakon of Norway, then residing in Buckingham Palace, visited the Hospital in 1943. Sister Mace, instrumental in establishing the relationship, received the Royal Norwegian Order of St. Olav in 1945.
- Warren MW. Care of the Chronic Aged Sick. The Lancet. 1946, 1:841-43.
  - The chronic aged sick was defined as “patients over 60-65, who partly because of age and partly because of the nature of their health complaint require long-continued treatment before being restored even to partial health.” Again, she advocated for them being cared for on “special blocks of a general hospital.” The aim of treatment remained returning home, but their hospital stays would be “measured in months, rather than weeks.” Warren wrote that admission to residential facilities should only occur through geriatric hospital units after “all possible treatment has been given, and the social background carefully studied.” The particular residential facility chosen should be in the best interests of the patient. She felt these requirements would likely change because of the future “larger proportion of patients … [coming] from the professional classes.” Her exhortations for an attitudinal change were strong. In a section titled *The Untreated Patient*, she wrote that the end result was a ”miserable state, dull, apathetic, helpless, and hopeless [as] life lingers on sometimes for years, while those around them whisper arguments in favour of euthanasia.” Compared to the 1943 publication, the classification system had evolved, and the required treatment team was elaborated on. There was a comment on the need for “kindly discipline” with patients.
  - Anonymous. Medical Women’s Federation. The Lancet. 1946, 1: 383-84.
    - A summary of a talk Warren gave to the London branch of the Medical Women’s Federation predating the publication that covered the same material and served as the base for the 1946 *Lancet* publication.
  - Anonymous. The Care of the Aged Sick. The Medical Officer. 1946, 76:45.
    - In this report of Warren’s address, the author stated that tackling this challenge could increase the “length of life beyond the point where usefulness can be maintained.”
    - This talk was also reported on in the April 1946 edition of the *Medical Women’s Quarterly Review*.
  - Warren MW: El cuidado del anciano crónicamente enferno. Salud Pública de México. 1996, 38(6):525-32. (This Spanish translation was published on the 50^th^ anniversary of the 1946 *Lancet* paper.)
- Warren MW. Geriatrics: a medical, social and economic problem. The Practitioner. 1946 (November), 157:384-90.
  - Warren began by noting financial disincentives for physicians to see older patients, which was balanced by a positive argument from a societal perspective for their better care. She wrote that “Nobody is more truly imprisoned than an elderly infirm person who has entered a hospital, and responding slowly, finds himself confined forever.” As an example of the excess disability that occurred when only custodial care was offered, she described 42 long-stay patients transferred to her unit in 1945. All were “bedridden [with contractures], doubly incontinent and helpless.” In three months, all were feeding themselves (none had before), 17 were sitting in chairs, 3 were walking with assistance, and 5 (12%) had been discharged (Warren later erroneously said 20% had returned home; reference - Anonymous. Medical Society for the Care of the Elderly. The Lancet. 1950, 1:861). She estimated that 30-35% of all admissions to a geriatric ward would return to the community, 40% would die, and 25% would remain as a “permanent residuum.” Warren felt all older medical and surgical patients (except for peri-operative care) entering hospitals should be cared for on geriatric units.
- Warren MW. Care of the chronic sick. 2. Clinical Aspects. Brit J Phys Med. 1947, 10 (March-April):36-38.
  - The benefits of rehabilitation for the chronic sick were not yet “fully recognized.” Acute from chronic disease as to their causes, duration, and sequelae were differentiated. She insisted older patients “must be treated as a whole.” The causes and consequences of the bedfast state were outlined while general advice was provided on addressing the psychological and functional needs of chronic patients. “So far all the methods of rehabilitation mentioned have been undertaken by the medical and nursing staff” but now was the time for the greater involvement of the “real experts” (physiotherapists and occupational therapists).
- Warren MW. A new outlook on the care and treatment of the elderly. Nursing Times. 1947, 43:544-46.
  - Warren was puzzled by the “strong disinclination” of nurses to work with chronic patients, which she felt needed to change. She called for additional nursing staff on geriatric wards compared to medical ones. In addition to managing physical needs, the “right psychological” approach was required and referred to the “defeatist” attitude held by many health care professionals. Warren gave “special thanks” to Sisters Bowen and Smythe as well as Miss Atkin from the occupational department, for their contributions to the work being done at the WMH.
- Committee on the Care and Treatment of the Elderly and Infirm (membership was Aitken JK, Amulree [Lord], Anderson AG, Brooke EB, Carling ER, Cosin LZ, Crothers EM, Esslemont M, Fenton J, Gordon RG, Green TA, Guttmann E, Kemball MMC, MacFeat G, Morris C, Newell RL, Rogers AT, Steel WD, Sturridge FR, Wand S, and Warren M). Report (1947) of the Committee on the Care and Treatment of the Elderly and Infirm. Brit Med J. 1947, 1 (Suppl):133-40.
  - The Committee recommended the gradual establishment of geriatric departments in general hospitals for the investigation and treatment of older patients. Large, stand-alone institutions for the chronic sick were “deplored.” Components of these departments were in-patient wards for the exclusive use of older patients, long-term annexes (either on or off site) under the medical supervision of the geriatric department (for the “20% residuum”), and residential homes operating “in close association” with the geriatric department. The functions of these departments included accepting new older patients whether acutely or chronically ill requiring hospital care, accepting transfers from other hospital wards, providing facilities for investigation and treatment, providing observation wards for the primary investigation of older psychiatric patients and their medical treatment, providing earlier and “more adequate and prolonged” rehabilitation, discharging rehabilitated patients and “resettling” them as necessary, arranging for prompt transfers of “irremediable” patients to long-stay annexes, assessing and periodically reviewing the suitability of everyone recommended for these annexes, offering general assistance to the coordination of medical and medico-social work for the “elderly sick”, providing on request advice to general practitioners on their older patients on home visits or in clinics, offering medical advice on the medical aspects of welfare or housing plans for older persons, teaching on the geriatric aspects of medicine/ nursing/ physiotherapy in select departments, and encouraging research on older patients. This recommendation was based on input from E.B. Brooke, L.Z. Cosin, and Warren who had written a draft of a paper on the topic they intended to publish (it never was).
  - Report also published as
    - Committee on the Care and Treatment of the Elderly and Infirm: The Care and Treatment of the Elderly and Infirm: Report of a Special Committee of the British Medical Association. British Medical Association (London), 1947. (28-page booklet.)
    - British Medical Association: When You Are Old. British Medical Association (London), 1947. (Pamphlet based on the Report written for and sold to the general public. Warren was acknowledged for her “valuable help” in preparing it.)
- Warren MW. The Evolution of a Geriatric Unit from a Public Assistance Institution, 1935-1947. Proc R Soc Med. 1948, 41(5):337-38.
  - This focused on the bed numbers of the WMH geriatric service. In June 1935 the WMH became responsible for 874 residents in Warkworth House. This included 16 maternity and about 144 “mental observation” patients excluded from any further comment. Of the remaining 714 more than half were bedridden, often for years. Two hundred “elderly and destitute able-bodied workers” were quickly transferred to Percy House. Between June 1935 and March 1936, the 514 remaining were examined and classified. Over the next three years the total number of beds was gradually reduced to decrease crowding and provide more therapeutic space. Equipment was obtained and wards upgraded. In 1939 two wards with 67 beds were transferred to other hospital services and another ward of 18 beds was converted to a gymnasium and x-ray department. The effectiveness and efficiency of the service gradually improved. By 1948 only a “compact geriatric unit” of 200 beds was required. G.F. Adams referred to this as the “substitution of 200 beds with a turnover for 700 beds with stagnation.” (Adams GF: Geriatrics in Northern Ireland. The Lancet. 1949, 2:1095-97.) Compared to other sources of information, there are inconsistencies on the specific numbers but consistency in the general trend described (MW Warren, *Geriatrics* 1948 – see below; Shaw P, Pam M: Obituary – Marjory Winsome Warren, C.B.E, M.R.C.S, L.R.C.P. Medical Women’s Federation Journal. October 1960, pp 197-98). By 1960 the bed number had further declined to 130.
  - Anonymous. A New Outlook in the Wards. The Lancet 1947, 1:760-62.
    - This provides details on the physical upgrade made to the wards, the process of care used, and the positive attitudes of staff and patients alike.
- Committee on the Care and Treatment of the Elderly and Infirm (membership was Aitken JK, Amulree (Lord), Anderson AG, Brooke EB, Carling ER, Cosin LZ, Esslemont M, Gordon RG, MacFeat G, Rogers AT, Steel WD, Sturridge FR, and Warren M). “The Right patient in the Right Bed” – First Supplement to the Report (1947) of the Committee on the Care and Treatment of the Elderly and Infirm. Brit Med J. 1948, 2 (Suppl):71-72.
  - The Committee felt their 1947 plan would help in addressing the then current challenge of an inadequate number of hospital beds. Additional measures proposed were short-stay hostels for patients undergoing investigations, “half-way houses” for hospitalized older patients no longer acutely ill but not fit for discharge to residential accommodation, and the expansion of out-patient services, which were conditional on the availability of transportation, establishment of regional advisory committees, and administrative coordination.
- Warren MW. Physical Medicine in Geriatrics. Brit J Phys Med. 1948, 11(6):167-69.
  - Older persons were more prone to degenerative disease and “accidents” (falls and fractures) than younger adults and took longer to recover (E.J. Stieglitz was quoted as stating the reparative process increases by 24 hours every 5 year of life). Management of the primary condition, prevention of complications, and treatment of secondary conditions all had to be addressed. A brief discussion on personnel and teamwork was included. Warren first described her bed-end exercises in this paper and the following one (1948 *The Medical Press and Circular)*. With a board placed across the foot of the bed to prevent slipping, the patient gripped the rail at the end of the old iron bedsteads then in use. First sitting and then standing they would raise one foot at a time. This was followed by standing from a chair while gripping the bed rail. Once mastered, the patient progressed to walking first across the bed-end and then away from the bed with aids and personal assistance. Bed-end exercises were called by Dr. G.F. Adams as “one of the most miraculous inventions of all time.” Warren felt you would be “wasting your time asking a patient to walk until they can stand and balance themselves.” (Professor George Adams CBE in interview with Dr. Max Blythe Oxford, 22 January 1996. The Royal College of Physicians and Oxford Brooke’s University Medical Sciences Video Archive MSVA 120, page 15.)
    - Dr. Ronald Bayne, a Canadian physician who trained under Warren in the 1950s wrote a short poem about bed-end exercises that he shared with me (personal communication) –

Bed ends, bed ends,

Prevents you getting dead ends;

So do not shirk,

But work and work!

It’s later than you think friends,

It’s later than you think.

- Warren M. Care of the Hemiplegic Patient. The Medical Press and Circular. 1948, 219(18):396-98.
  - Hemiplegia can be due to cerebrovascular disease (most common cause), trauma, or a tumour. These patients in the past were considered irremediable with little if any rehabilitation attempted. General principles for their care that she proposed included a full clinical assessment to ascertain “cause, site and extent of the lesion”, prevention of complications, and rehabilitation. As soon as the patient was conscious, reassurance and encouragement should be offered. Warren advised keeping most hemiplegic patients on bedrest for at least 4 weeks. Bed-end exercises slightly modified for a hemiplegic patient were recommended. “Satisfactory progress” in rehabilitation typically occurred at three months though it might take 10-12. After discharge, regular follow-up was recommended.
- Warren MW. The Evolution of a Geriatric Unit. Geriatrics. 1948, 3(1):42-50.
  - Initially the geriatric unit was “regarded by most of the medical staff as a convenient unit to which to transfer all their unwanted patients … usually without consultation.” This slowly changed, and by 1948 admissions were restricted to older patients from the community or transfers from other hospital units after consultation. The commonest presentations encountered were “general debility”, psychiatric conditions (including dementia), cardiovascular disease (including stroke), arthritis, “nervous” diseases, chest conditions, neoplasms, and injuries (as transfers from orthopedics for rehabilitation). During the early years of Second World War there was a decrease in admission requests, but this later reversed. In 1944 two wards (one female, one male) began record keeping. Between 1944-46 a total of 1364 patients were admitted to them. A total of 589 (43%) died, 349 (26%) were discharged home, 137 (10%) went to a residential home, and 289 (21%) remained as a “residuum.” No comparison data were provided. The unstated assumption was that few if any would have been discharged to the community prior to the development of the geriatric unit. Updated figures for the entire unit and/or from a more stable (or normal) period were never provided. These results were virtually identical to those published by L.Z. Cosin for the Orsett Lodge Hospital geriatric unit. From 1944 to 1947 mortality was 38%, discharges 37%, those still on the unit 21%, and 4% had been transferred (Cosin L: A Statistical Analysis of Geriatric Care. Proc R Soc Med. 1948 May; 41(5):333-6). The need for more physiotherapy, the value of social worker input, and a plan to establish an out-patient service were also noted in this paper.
  - The control of admission to geriatric units proved to be contentious. This irritated medical staff trying to discharge unwanted patients, but broader ethical issues were also raised. Dr. A. Elliott, a County Medical Officer, wrote that having all beds for the aged chronic sick follow the WMH practice of the service controlling admission would lead to “those cases clearly not able to benefit therefrom to shift as best they may – and maybe die as quickly as possible – then, I am afraid, we are well on the way to that ‘deterioration of ethics’ which I referred to” (Elliott A. Nurses for the Elderly (Correspondence). The Lancet. 1947, 2:71).
- Warren M. The Medical Care and Rehabilitation of Elderly People. The Advancement of Science (London). 1949, 6(21):18.
  - This was a summary of a talk Warren gave at a meeting of the British Association for the Advancement of Science. Warren felt acute geriatric units, long-stay annexes, and associated residential facilities should be under the medical supervision of geriatric departments and voiced displeasure with the division of medical and social care that had taken place in England.
- Warren MW. The Role of a Geriatric Unit in a General Hospital. Ulster Med J. 1949, 18(1):8-10, 11-17.
  - The care of the “elderly sick” required “radical reform” if “the burden to the community is not to become intolerable, or the total sum of misery unbearable.” Large, isolated and under-resourced hospitals for the chronic sick should be replaced by geriatric units in general hospitals linked with long-stay annexes and residential homes. These units would be primarily responsible for acutely ill older patients as well as those with chronic diseases. Admissions would come directly from the community or taken in transfer from other hospital departments after consultation - “chiefs of other units will no longer expect them [the geriatric unit] to accept all long-term sick, and particularly those whom they want to pass on.” Over 80% of patients admitted were for degenerative arterial disease (causing mental deterioration, peripheral gangrene and strokes), cardiac disease, arthritis, malignancies, progressive neurological conditions, and/or “lack of social care.” Assessment and treatment should address both medical and social issues. To integrate medical and social services, a regional committee with executive powers was proposed.
- Warren MW. Chronic Aged Sick. In: The Medical Annual 1949 (edited by Sir H. Tidy and A. Rendle Short). John Wright and Sons Ltd. (Bristol), 1949, pp. 70-76.
  - This overlaps with the preceding paper. Some sections are nearly verbatim. The 1947 *Committee on the Care and Treatment of the Elderly and Infirm* report was referred to as the basis for planning a geriatric service. Advantages of geriatric units were “patients more often make congenial contacts with each other and definitely stimulate each other in a healthy spirit of rivalry” plus greater opportunities to train staff, equip the wards, and perform clinical research. Team staffing needs were for physicians, nursing staff, social workers, physiotherapist, occupational therapists, and the “services of a dietitian and chiropodist.” Warren proposed establishing “experimental homes and units so that all methods can be given a full trial before there is any attempt to standardize.”
- Warren MW. Protracted Illness – Management and Rehabilitation. In: British Surgical Practice, Volume 7 (edited by Sir Ernest Rock Carling and Sir James Paterson). Butterworth & Co. (Publishers), Ltd. (London), 1950, pp. 177-84.
  - Protracted illnesses were defined as “conditions which need continuous treatment for long periods of time before recovery can be established.” They were most commonly encountered in older patients. Areas covered in the paper included post-operative care, general approach, “psychological care” for patient “anxieties”, sequelae of prolonged bed rest, and pain management for incurable patients. With pain medications, it was “essential for the patient’s well-being that the dosage be increased as slowly as possible, using the least potent drugs first.” For malignancies she suggested starting with ASA or phenacetin (this agent was later withdrawn from use due to its carcinogenic and kidney-damaging properties) at low doses taken initially as required and then regularly. When no longer adequate, she proposed escalating first to codeine and then morphine or Dover’s powder (no longer in use). Hypodermic injections of morphine would be an option when the pain was only controllable by this means. In this manner, “the choice of drugs, their dosage, and their frequency is controlled individually by the patient under the careful supervision of the staff.”
- Warren MW. Gerontology. In: The Medical Annual 1950 (edited by Sir H. Tidy and Mr. A. Rendle Short). John Wright and Sons Ltd. (Bristol), 1950 pp. 120-24.
  - Warren thanked Dr. M. Peszczynski for his assistance in this review of recent literature.
- Warren MW. Geriatrics. In: Recent Advances in Physical Medicine (edited by Francis Bach). The Blakiston Company (Philadelphia), 1950, p. 314-29
  - In addition to being more likely to need rehabilitation due to chronic conditions and “accidents” (falls and fractures), older patients required more recovery time and measures to prevent secondary disabilities. Short, frequent rehabilitation sessions were recommended because of “general frailty” and other factors found in older patients. The use of simple interventions would allow nursing staff or the patient to conduct sessions between physiotherapy visits. The bulk of the paper was given over to general principles (i.e., aim to maintain or restore mobility and independence, gain the patient’s confidence and co-operation, and create a hopeful atmosphere), dealing with bedrest, and specific recommendations for arthritis, stroke, femoral neck fractures, and other conditions.
- Warren MW. The Role of the Social Worker in a Geriatric Service. Mother & Child. 1950, 20:346-51.
  - A good part of the paper deals with how social workers can help prevent disease and avoid hospitalizations. When an older patient is hospitalized, social workers play a vital role in “keeping the social and environmental picture of the patient before the medical staff, when helping to plan with the physician for the patient’s future.” Though not mentioned, her sister Enid, who lived with Warren, was a distinguished social worker (A Tribute from Colleagues and Friends. Portrait of a Social Worker – Enid C. Warren O.B.E., 1903-1980. British Association of Social Workers, 1982; Baraclough J: Warren, Enid Charis (1903-1980, medical social worker. Oxford Dictionary of National Biography (Oxford University Press) 2004, 57:477-78).
  - Rosin AJ. The Doctor-Relative Relationship. Social Work in Health Care. 1976, 1(4): 499-505.
    - Rosin describes “relatives” clinics that were developed by Warren at the WMH. They consisted of a joint interview between a relative and a junior or senior physician followed by an interview of the family by a social worker. These interviews were in turn followed by a joint discussion between the physician and social worker. Though time consuming, they were helpful for patient care and instructive for junior physicians.
- Warren MW. Activity in Advancing Years. Brit Med J. 1950, 2(4685):921-24.
  - Warren wrote that “… in treating the elderly, rather than deciding how soon they may safely get up, one should consider very carefully whether it is really necessary and wise to put them to bed” while “nothing that a patient can do for himself should be done for him.” The effects of inactivity were reviewed. Treatment should be undertaken by a team whose members “must work in complete harmony if full success is to be achieved.” Specific recommendations for arthritis and hemiplegia were included.
- Warren MW. The Facilities Available for the Care of the Elderly in the Home. The Practitioner. 1950, 165(986):156-63.
  - Warren questioned the admission to hospital of an “elderly invalid” when this was not “really necessary on medical grounds” or entering a welfare home when it was possible for them to remain home. Older persons should be kept at home whenever possible (including when ill if sufficient assistance was available) with relatives and friends called upon first for help. She noted that the number of older persons seeking admission to an institution exceeded vacancies. She partly blamed this on “irresponsible relatives” not willing to assist older family members. Available domiciliary resources included relatives/ friends, hospital-based consultants, monetary grants from the National Assistance Board, local authority services (i.e., health visitors, district nurses, home help, services for the blind, medical aids, welfare), and assistance from voluntary agencies. The latter varied widely making it difficult for general practitioners to know what was locally available. She ends with “two golden rules … first, to put the patient into touch with the correct service or services, and secondly, to use the services carefully and providently.”
- Warren M. The Elderly in the Community. Social Service Quarterly. 1951, 24(3):102-6, 120.
  - Warren felt “the greatest challenge of our time is the maintenance of an ageing population in health and contentment integrated within the population in such a way as to support those who need help without undue burden upon others.” Though most older persons were doing well, it was a time of “waning strength … [that coincides with] loss of employment and reduced income.” Overall “few escape without some physical frailty, many suffer some psychological shortcomings and the majority need some personal service.” To prevent health issues, she suggested avoiding overwork, fatigue, and “indiscretions of all kinds and living by regular habits.” Older persons “should never allow anyone to do for him what he can do for himself.” At a community level she lamented the lack of an overall plan for many older persons “not sick enough to justify admission to a hospital, and yet too disabled or frail for a vacancy in a Home.” She ends by warning of the harm done “by under-treatment and an attitude of defeatism.”
- Warren M, Gumpert M, Haarlöv T, Havighurst RJ (Chair). The University of Chicago Round Table – American Problems of Old Age Part II: Living Arrangements for the Elderly (Program No. 703). University of Chicago (Chicago), September 16/ 1951, 11 pages.
  - This is a transcript of the September 16^th^, 1951 broadcast of an American radio series consisting of informal roundtable discussions concerning public issues. The panel concluded “that the one-generation pattern” of housing with “fairly close social contact with other people in the community and with his family members” was the most desirable living arrangement for older adults.
- Warren MW. Geriatrics. In: The Medical Annual 1951 (edited by Sir Henry Tidy and A. Rendle Short). John Wright & Sons (Bristol), 1951, pp 108-112.
  - Warren saw little merit in conducting surveys of patients showing problems and “then leaving matters status quo.” To provide “good medical care” practitioners needed more knowledge on senescence, its differentiation from pathological states, the psychology of old age, treatment options and likely results, and available resources. Senescence was viewed as normal and consisted of a general and gradual “’running down’ of the human organism.” Aims of medical care were to prevent disease whenever possible, reduce medical disability to a minimum, obtain and maintain maximum independence, and teach the patient to adjust themselves to any residual disability. The challenges in diagnosing older patients and common presenting symptoms were reviewed. Post-diagnosis treatment plans should have realistic goals. She closed by emphasizing the need for better management of strokes.
- Warren M. Growing Old Gracefully. Health Horizon. 1951 (Summer Issue):26-31.
  - Warren wrote that “misfits and maladjustments” mainly occurred among adolescents and older persons as both are times of “great insecurity of place.” “To be at their best” older persons required good health (it is “easier to keep well than get well”), useful occupation appropriate for their health status (“while an individual is working, he is carrying out man’s function, as soon as he is idle he becomes introspective and a parasite”), maintaining one’s appearance (“Let us take heed how easy it is to lose charm (and therefore caste) and yet how much better to remain attractive and therefore acceptable”), developing “traits of character which are most acceptable to others”, housing security, independence, and friendship.
- Warren MW. A Geriatric Unit in a General Hospital – England. J Gerontol. 1951, 6 (Suppl 3):166. (Abstract)
- Warren MW. Planning Housing for the Aged (England). J Gerontol. 1951, 6 (Suppl 3):166. (Abstract)
- Warren MW. Rehabilitation of the Hemiplegic. J Gerontol. 1951, 6 (Suppl 3):166-67. (Abstract)
- Warren MW. Voluntary Services for the Elderly in Great Britain. J Gerontol. 1951, 6 (Suppl 3):167. (Abstract not received by the time the program went to press.)
- Warren M. Rehabilitation of Geriatric Cases. Brit Med J. 1951, 2:238-39. (Correspondence)
  - A device to straighten the leg in spastic hemiplegia and a three-legged walking stick (the *Warral*) designed by her and Leslie Ralph of Remploy Limited were described.
- Warren M. Convalescence for the Elderly Patient. Ment Health (London). 1951, 10(3):68-69.
  - The WMH geriatric unit was provided access to a holiday home in Bognor for two months during a winter that was used with benefit by 8 patients.
- Warren MW, Kaminski J. The Elderly Arthritic Patient. Rheumatism. 1952, 8(4):74-82.
  - The commonest condition seen in old age was arthritis. While “many may be only slightly handicapped, a large number are crippled and seriously disabled.” Both authors felt there was “too defeatist an atmosphere concerning the treatment of arthritis and too many quack remedies on the market.” In an unnamed geriatric unit (presumable the WMH) 5.9-8.3% of annual admissions over a five year period were primarily because of arthritis. In many more arthritis was a secondary condition. These admissions were accounted for by osteoarthritis (58%) and rheumatoid arthritis (42%; most cases were “burnt out”). For arthritis of the knee, limited lateral excursion of the patella was deemed the most useful sign in assessing prognosis. Management was divided into local (i.e., prevention of contractures, exercise, occupational therapy, physiotherapy, use of “gadgets”, a term then used for medical appliances; reference - National Association for the Paralysed: Gadgets. The Grange Press (Southwick, Sussex), 1953), general (i.e., dealing with co-morbidities and pain), and social therapies focusing on residual disabilities.
- Warren MW, Kountz WB, Pratt LA, Stieglitz EJ (Chair). Geriatric Medicine: Therapeutic Aspects. J Gerontol. 1952, 7(1):100-15.
  - This lively panel was held during the Second International Gerontological Congress and Fourth Annual Scientific Meeting of the Gerontological Society of America. Warren felt therapy depended primarily on an accurate diagnosis including, with the assistance of an informant, the older person’s psychological and social state. She strongly cautioned against blaming advanced age for health issues particularly when there had been an abrupt change. Dealing with multiple diagnoses, the dangers of inactivity, and “defeatism” on the part of the patient were brought up as other important considerations. No older patient “is to have done for him anything he can do for himself.” In enforcing this she would “insist on their cooperating and even take the part of … irritating them” if needed. When asked whether she would use “therapeutic rage as a stimulant”, she responded yes. There should be “no prejudice” in therapeutics because of age. Though few chronic conditions can be cured, “the large majority can be remedied and improved” with the patient taught to “live intelligently with his residual disabilities.” While geriatrics began before birth academically, from a therapeutic standpoint it started around 50. When asked about sedatives, Warren said she would use phenobarbital with “extreme care” in small doses due to the sensitivity of older persons to drugs. The other panelists strongly disagreed with her.
  - A 1957 letter to the *British Medical Journal* raised concerns about the “tendency to stimulate elderly patients who are in the terminal stages of an illness to make efforts which are sometimes beyond their powers and certainly beyond their inclinations … it seems to me this stimulation amounts almost to bullying and is not so much being cruel to be kind as being cruel to no very good purpose.” There is nothing to indicate that this letter was written specifically about Warren, but it does indicate the fine line between encouraging and bullying patients. (Loring JN. Stimulating the Elderly (Correspondence). Brit Med J. 1957, 2: 1437)
- Warren MW. Geriatrics: Care of the Elderly in their Homes. In: Medical Annual 1952 (edited by Sir Henry Tidy and A. Rendle Short). John Wright & Sons (Bristol), 1952, pp.155-58.
  - Hospital admissions should be limited to those who require them, but if an older person needed one it should be quickly available. She lamented a “tendency on the part of some sections of the community to claim a bed in hospital and then to try and keep it because of the comfort and service which it provides.” She felt this occurred most often in older patients as younger ones had more incentives to return home. She described resources that supported older people at home such as health visitors, district nurses, home help, medical devices, and voluntary help. Warren reiterated keeping people in bed for as short a period as possible. If admitted to hospital, “continuity of medical planning between the doctor in hospital and the doctor who will carry on treatment outside” was needed. She cautioned physicians against attributing abrupt changes in an older patient to age alone, noting that clinical presentation of conditions like coronary thrombosis are not as “spectacular” in older patients compared to younger ones, and that presentations can be non-specific.
- Warren MW. Retraining the Elderly Hemiplegic. Geriatrics. 1953, 8(4):198-203.
  - After painting a glum picture of the state of untreated hemiplegic patients, Warren stated physician needed to “consider treatment of the psychological and physical condition” of these patients. For the initial evaluation of an older hemiplegic patient Warren recommended a full clinical examination, search for a neoplasm (because of the possibility of a malignant etiology), lumbar puncture, and blood work. Treatment should begin as soon as possible. Psychological interventions, which should begin as soon as possible, included reassurance and encouragement while physical measures would include both preventive (positioning, range of motion) ones and active rehabilitation. Patients should “never have done for him anything in the way of self-care that he can do for himself” or “be touched or handled unless it is absolutely necessary.” Exercises were divided into “those which are done in bed, on the bed, at the bed end and away from the bed.” Bed-end exercises were of the greatest importance, as “unless he can be taught to stand in this way he cannot learn to walk.” Rehabilitation should continue “until maximum results are obtained.”
- Warren M. The Care of the Chronic Sick. Tech Hosp Med Soc Sanit. 1953, 8(92):26-30.
- Warren MW. Incontinence in Old People. In: The Medical Annual 1953 (edited by Sir H. Tidy and A. Rendle Short). John Wright and Sons Ltd. (Bristol), 1953, pp. 215-19.
  - Urinary incontinence was commoner in women and both isolated urinary and combined incontinence were more common than isolated fecal incontinence. “Retention of feces with overflow” was an under-recognized cause of fecal incontinence. In management the “patient as a whole” had to be considered. Warren reviewed what was then known about the control of micturition and defecation. She stated both urinary and fecal incontinence were “all” due to loss of inhibition or “local causes increasing the excitability of bladder or rectum.” Notwithstanding this statement, Warren then described additional physical and psychological causes (see 1956 *Treatment and Care of Elderly Patients* for another classification scheme she proposed). An “inadequate personality” was the most common contributing psychological factor. Treatment entailed dealing with local factors if present. If due to a loss of inhibition, it was important to differentiate between “structural damage” and “loss of function only.” The paper ended by noting incontinence may arise during a period the patient was bedfast, which should be avoided if possible.
- Warren MW. Nursing care of the incurable patient - 1. Nursing Mirror. 22 October 1954, 100:251-52.
  - This was first of two articles based on a lecture she gave at the Professional Nurses and Midwives Conference that year. “Incurable patients” included the “blind, the deaf, the crippled, the physically handicapped and the mentally weak”. Warren felt this definition would include “most old people” with “multiple pathology” who “carry with the new disabling condition, the scars and assaults of earlier days.” If not “obviously in a terminal condition”, an optimistic approach was essential. She advocated actively treating all disabilities present and preventing the development of additional ones.
- Warren MW. Nursing care of the incurable patient - 2. Nursing Mirror. 29 October 1954, 100:319-20.
  - The principles of caring for an incurable patient were treating as fully as possible, preventing secondary conditions/ complications, and training the patient “to live as independently as possible and intelligently with his remaining disabilities.”
- Warren MW. Cerebrovascular Disease in Old Age. In: The Medical Annual 1954 (edited by Sir Henry Tidy and R. Milnes Walker). John Wright & Sone (Bristol), 1954, pp. 89-93.
  - Warren’s classification for cerebrovascular disease included patients who “experience momentary loss of consciousness” with no residual disability (i.e., syncope). She felt it was “generally unwise, and often unkind to reveal the presence of hypertension to the patient, and often it is better not to discuss it even with relatives.” For hypertensive patients presenting with syncope, she advised rest, light diet, weight loss, and phenobarbital twice daily. If there were residual disabilities, reassurance of the patient and rehabilitation based on a recovery plan of 6-9 months (perhaps longer) duration were recommended. The patient “must be persuaded to help himself by accepting advice.” Cerebrovascular disease was estimated as accounting for 20% of all admissions. Warren ended by describing “complicated cases” requiring longer and more specialized therapy. She estimated cerebrovascular disease accounted for about 20% of admissions to her unit.
- Warren M. Problems of Old Age. Brit Med J. 1954, 2(4898):1212. (Book review)
- Warren M. Physical Agents in the Treatment of Chronic Patients. In: The Medical Annual 1955 (edited by Sir Henry Tidy and R. Milnes Walker). John Wright (Bristol), 1955, pp. 294-305.
  - Paraphrasing Karl Marx, she wrote “… the individual [should] receive what he needs and be in a position to give what he should.” Here Warren reviewed physical agents for the relief of “invalidism and disability.” She felt a “really good medical service to-day should no longer be assessed purely on the grounds of cure, but should be fully reviewed as to the degree of disability which can be overcome.” Warren addressed clothing, heating, lighting, home design, and then moved on to medical and surgical appliances such as a tetrapod, tripod, and shuffle board for bed to chair transfers. She claimed to have designed all three. In another publication from persons working on the WMH unit, the idea of the shuffle board was credited to a patient cared for on the geriatric unit (Vinden K, Wayne JRD: Aids for the Disabled: Bridging the Gap from Bed to Chair, Brit Med J. 1955, 2: 432). Wheelchairs were also briefly mentioned. Warren influenced their design and use in post-war Britain (Woods B, Watson N: When wheelchair innovation in Britain was under state control. Technology and Disability. 2005, 17:237-50). She ended by noting the need for patient education and maintenance of ultimate physician responsibility for their use.
- Warren M. Problems in Connection with Nutrition of Elderly People. Int J Food Sci Nutr. 1955, 9(4):154-58.
  - Both under- and over-feeding were covered. Physical (teeth, senses, “physical infirmity”), psychological (“food fads”, emotional state), and social (housing, shopping access, finances) factors influencing nutrition were reviewed and general advice given. Warren viewed dietitians as “essential members of a good and full therapeutic team.”
- Warren MW. Geriatric Medicine. J Gerontol. 1955, 10:188. (Book review)
- Warren M. Health of the Ageing. Brit Med J. 1955, 2(4930):28. (Book review)
- Warren MW. A Shared Responsibility. The Lancet. 1955, 268(6865):673. (Correspondence)
  - Warren pointed out that “responsibility for patients already in our charge is in no way lessened by the necessity to admit others” and wrote against the practice of “premature or precipitous discharge.”
- Bayne JR, Warren M. Disposal of the Chronic Case. The Lancet. 1955, 268(6878):1317.
  - This case report warned of the dangers of labeling a patient as “irremediable and beyond therapy.” In 1954 a 74-year woman was transferred to the geriatric unit. She was born with left sided weakness and later deemed mentally unstable though at the time of transfer there was no evidence of the latter. Over the years she became estranged from her family. In 1935 she entered hospital for pneumonia (successfully managed), refused a recommended left lower extremity amputation, and then was kept in against her wishes. In 1940 she was evacuated from London (where she had lived her entire life) and admitted to a “chronic-sick hospital in the provinces” where she stayed until 1954. Though treated with “greatest kindness”, she was refused rehabilitation. After transfer she underwent a comprehensive evaluation, orthopaedic surgery, and rehabilitation on the geriatric unit. She returned to the community “after twenty years in hospital and thirty weeks of active therapy.”
- Warren MW. The Home Nursing of the Aged Sick. The Practitioner. 1955, 174(1043):567-73.
  - The “modern approach … [is based on the recognition] that there is no necessity for the aged to become bedfast and entirely dependent on others.” Nurses should focus on what required their skill and training, as much of the support an older person at home needs can be done by others. While older persons should have ready access to a hospital bed when needed and not be nursed at home (and deteriorate) because it was less expensive, determination of the best approach depends on the patient’s medical condition, “temperament”, social and physical environment, scope of domiciliary services available, and views of their general practitioner. Detailed instructions were provided for nursing an older person at home with pictograms on how to get a patient out of bed and the right way of sitting in a chair and transferring from a wheelchair to a bed. Warren concludes by stating home nursing was an “essential part of any good health service.”
- Warren MW. The Management of the Elderly Double Amputee. In: Old Age in the Modern World – Report of the Third Congress of the International Association of Gerontology (London, 1954) (edited by Dr. C.A. Boucher, Dr. O. Olbrich, Mr. W.A. Sanderson, Dr. J.H. Sheldon, Prof. R.E. Tunbridge, and Dr. Marjory Warren). E & S Livingstone Ltd (Edinburgh and London), 1955, pp. 562-70.
  - During the previous 5 years Warren had cared for 18 double amputees on the geriatric unit of the WMH. A total of 10 had been discharged, 6 were still in hospital, and 2 had died. She favoured pylons with curved rocker foot-pieces measuring 60 cm from belt to floor rather than prosthetic limbs for re-ambulation. Best results occurred when treatment was “undertaken in an atmosphere of optimism and healthy rivalry.” Warren acknowledged the contributions of Drs. M.M. Pam and J. Kaminski.
- [Partial reference] Warren MW. The Geriatric Approach. In: European Exchange Seminar on Social Services for the Aged. United Nations (Geneva), 1956, pages unknown.
  - I was unable to obtain a copy of the publication.
- Warren MW. Geriatrics. In: The British Encyclopedia of Medical Practice 2^nd^ Edition – Medical Progress and Cumulative Supplement (Edited by Sir Henry Cohen). Butterworth & Co, Ltd. (London), 1956, pp. 90-101.
  - In this literature review of recent developments, subjects covered were general principles, physical handicaps, independence in personal needs, and psychiatry in geriatric medicine (she cautioned against hasty certification and transfer to a mental health facility). Warren emphasized the need for flexible, practical and team-based care that included addressing the patient’s morale. The overarching goal was as much independence in personal care as possible. For confusion Warren advised addressing underlying physical causes and “better and more understanding nursing care, and maintenance of ‘diurnal activity’ and interest.” She commented on geriatric services (including a comparison of the respective merits of universal versus selective domiciliary visits), policy (speaking against “arbitrary retirement at the present chronological age” and for modifying the working conditions of older workers), the role of nursing and ancillary services, and research. She also noted that “It is frequently argued that geriatrics is not a specialist branch of medicine of medicine and with this view the majority of physicians, working in the field, would agree.” Dr. Kaminski was thanked for his assistance.
    - In the article she referenced a talk on *Speech Therapy in Geriatrics* that she gave July 22-26, 1955, at the Conference of the College of Speech Therapists held at Bedford College, London, England. Her publications included ones directly addressing the roles of dietitians (1955 *Int J Food Sci Nutr*), occupational therapists (1959 *Rehabilitation*), and social workers (1950 *Mother & Child*) in geriatric programs. Physiotherapists were included as members of the therapeutic team but at this time they primarily under the jurisdiction of the WMH Orthopedic Surgery Department (Matthews DA. Dr. Marjory Warren and the Origin of British Geriatrics. J Am Geriatr Soc. 1984, 32(4):253-58), which may have restrained her from writing about their roles. Many of her publications dealt with the responsibilities of physicians and nurses.
- Warren M. Geriatric Medicine. In: The Medical Annual 1956 (edited by Sir Henry Tidy and R. Milnes Walker). John Wright & Sons Ltd. (Bristol) 1956, pp. 168-174.
  - In this review of recent literature, Warren thanked Dr. Kaminski for his assistance.
- Warren MW. Treatment and Care of Elderly Patients. Hospital and Social Service Journal. 1956.
  - This 41-page publication was a collection of articles that previously appeared in the *Hospital and Social Services Journal* and was targeted to nurses in training. Topics covered were general principles, pressure injuries, incontinence, contractures, arthritis, accidents and amputations, hemiplegia, personality problems, and irremediable patients. She commented on something being “fundamentally wrong” if there was a lack of interest in nursing older patients, where a combination of a “humanistic and scientific approach” was needed. Warren felt all nursing students should be trained on geriatric wards. In the incontinence section the classification scheme used divided causes into physical and psychological ones. Under physical she listed “apparent” and “real.” The latter were due to loss of inhibition from central lesions (regular toileting was suggested for this) and local conditions (e.g., infections). “Apparent” included urgency, overflow and other reasons for not reaching the toilet in time (e.g., limited mobility, not aware where the bathroom was located). Stress incontinence was not mentioned though known at the time (Wilson TS: Incontinence of urine in the aged. The Lancet. 1948, 252(6523):374-377). In the accidents and amputations chapter Warren provided an overview of the commonest causes of falls. Personality problems dealt with older patients who were “antisocial in habits or unable to take care of himself by reason of forgetfulness, suspicion, apathy, disinterest or mental confusion.” She commented on the interplay between the person’s physical state and environment. The proper care of the irremediable patient required treating the whole patient, encouraging independence, and respecting their “amour-propre” (self-esteem).
- Warren M. Les altérations mentales chez les gens agés. Tech Hosp Med Soc Sanit. 1956, 11(125):30-31.
- Warren M. Le rôle d’une unité gériatrique dans un hôpital général. Tech Hosp Med Soc Sanit. 1956, 11(125):37-38.
- Warren MW. The Health of the Elderly at Home. Health Education J. 1956, 14(1):54. (Book review)
- Warren M. Treatment of Hemiplegia of an Old Person in a Private Home. Geriatrics. 1957, 12(4):267-68.
  - In the *Queries and Therapeutic Notes* section, Warren responded to five questions. For one, she stated on average “the patient is out of bed about ten to fourteen days after the incident”, shorter than what she recommended in her 1948 *Medical Press and Circular* contribution.
- Warren M. Modern Trends in Geriatrics. Health Education J. 1957, 15(4):263-64. (Book review)
- Warren M. Some Principles in Geriatric Medicine. In: Fourth Congress of the International Association of Gerontology (Merano [Bolzauo], 1957), Volume II. Tipografia Tito Mattioli (Fidenza, Italy), 1957, pp. 538-43.
  - Warren pointed out the “importance of attention to small things when treating elderly patients”, the necessity of treating the patient as a whole, developing a care plan, utilizing a full therapeutic team (consisting of physicians, nurses, social workers, and ancillary workers such as physiotherapists, occupational therapists, speech therapists, dietitians, chiropodists and others all working under the guidance of a physician), considering environmental conditions, and providing continuity of care. The physician was responsible for the care plan. If there was a difference of opinion between therapists and the physician, the physician “should explain carefully the underlying rationale and why such a regime is best and this must be then followed.” Warren felt much of the failure to achieve results in the rehabilitation of older patients was due to a lack of meticulous attention to detail.
- Warren M. The Problem of the Aged Amputee. Postgrad Med J. 1957, 33(383):436-43, 451.
  - Older patients with peripheral arterial disease also have a “variety of arteriosclerotic manifestations … [and] multiple pathology”, which complicates their care. Warren again voiced her belief that “most amputees, single and double, should be educated to walk again using a single pylon or two rocker pylons.” She felt moving on to prostheses should be “the exception rather than the routine.” Detailed advice for treatment phases (pre-operative care, post-operative and pre-pylon care, post-pylon care, maintenance and follow-up care) was provided. Drs. M.M. Pam and J. Kaminski were thanked for their help.
- Warren MW. Housing Needs of Old People. The Municipal Journal. 1957, 65: 405-406.
  - In an address Warren gave to the Royal Institution of Charted Surveyors, she said that inadequate access to housing was having a deleterious effect on welfare and hospital services. She provided recommendations (often very specific) on the site of housing, rooms, furnishings, location of shelves/ windows/ switches, specifics on doors/ sinks/ baths/ toilets, and both lighting and heating. Warren felt a survey of the number of older persons needing housing, “bold and imaginative research into the best kinds of housing for old persons”, and greater social work access for those at home were required.
- Warren M. Gerontology – A Modern Approach to Old Age. In: Aging is Everyone’s Concern - The Proceedings of the First Ontario Conference on Aging. University of Toronto (Toronto, Ontario), 1957, pp. 180-196.
  - These proceedings included a plenary address given by Warren. Taking a societal perspective, she dealt with changing demographics, maintenance of health, and retirement. Warren then focused on clinical issues and services. Warren concluded with “Those who live with, or work with or for, old people should neither patronize nor penalize them because of their age, nor yet should they scold or get sentimental about them.” What was needed is an “atmosphere in which self-esteem can thrive and all the old people’s assets and advice can be used.”
- Warren M. The Social Aspects of Geriatric Care. In: The Matrix of Medicine – Some Social Aspects of Medical Practice (edited by Nicolas Malleson). Pitman Medical Publishing Company, Ltd. (London), 1958, pp. 121-41.
  - Other than rearranging sections and relatively minor additions or deletions of text, this book chapter duplicates much of her contribution to the 1957 *Proceedings of the First Ontario Conference on Aging*.
- Bayne JR, Warren MW. The Hemiplegic in Hospital. Can Hosp. 1958, 35(2):50-52, 90.
  - The outcomes of all hemiplegic patients admitted to the West Middlesex Hospital in 1949 were presented. The majority of cases were admitted directly to the geriatric unit. Of the 131 patients 76 (58%) died, 43 (33%) were discharged home, 8 (6%) went to a welfare home, and 4 (3%) were transferred. Justification for publishing nearly 10 year old data was not provided.
- Warren M. Ageing on the Factory Floor. Occup Environ Med (formally British Journal of Industrial Medicine) 1958, 15(4):302-3. (Book review)
- Warren M: General Geriatric Problems and Medical Rehabilitation. In: Conquering Physical Handicaps: Official Proceedings of the First Pan-Pacific Rehabilitation Conference Held in Sydney, Australia, Nov. 10-14, 1958. Australian Advisory Council for the Physically Handicapped (Sydney, Australia), 1958, pp. 451-59.
  - The paper began with definitions, a review of the increasing proportion of older persons in society (with reasons why), and a description of sociological changes that made responding to this demographic shift challenging. It was followed by addressing clinical considerations. Every older person when seen for a health issue should have their presenting condition or complaint addressed, an evaluation of the “whole patient” done, and their social condition considered. Management required active preventive and therapeutic interventions. Rehabilitation methods should be simple and able to be carried out concurrently with medical treatment in order to prevent further deterioration and save time. Sessions should be frequent, short, and done in a standardized manner. Warren felt through “continuity and undivided medical leadership, all aspects of the illness *and* of the individual will be kept under critical and constant supervision.” Warren advised geriatricians not to be “sensitive to the taunts of the cynic, or unduly depressed when death cheats him of success.” She acknowledged what she had learned from colleagues and older patients “whom it has been my privilege to serve.”
- Warren M. Research on Ageing. Brit Med J. 1958, 1(5076):933-34. (Book review)
- Warren MW. The Clinical Management of Apoplexy. In: Proceedings of the Second Meeting of the European Clinical Section of the International Association of Gerontology, Nancy-Vittel, 1959, pp. 67-71. (Not able to obtain a copy.)
  - Also referenced as being published in the *Rev franc Geront* 1959, 5:417-23.
- Warren M. The Principles of Geriatric Medicine. Bulletin of the Post-Graduate Committee in Medicine, University of Sydney. 1959, 15:55-62.
  - Given as a lecture during the 1958 Annual Subscription Course held in conjunction with the Pan-Pacific Rehabilitation Conference, Warren said the care of older persons required a fully integrated system consisting of general practitioners, medical officers of health, and hospital services. She felt the preventive side was “more important than the therapeutic” and that older persons should be cared for at home whenever possible. The bulk of the paper covers “how elderly patients reach a doctor, the characteristics of illness in old age, and the reactions of old persons to deterioration in health.” Many are brought in by, for example, a relative or friend. She felt it was important to determine their motives, as they could vary from true concern to a “desire to get the old person ’admitted somewhere.’” Patients should be assessed “as a whole” with a full medical and social history coupled with a physical examination. Disease in old age was usually degenerative, often multiple, insidious at onset, progressive, and marked by slow recovery. Presenting complaints were often vague. Clinical pearls for the commoner ones were given. Older persons might react to illness with feelings of insecurity, depression, frustration, apathy, and fear.
- Warren MW, Vinden KE. The Place of the Occupational Therapist in the Geriatric Unit. Rehabilitation. 1959, 29:15-19.
  - Vinden was the head of occupational therapy (OT) at the WMH. Physicians were now acknowledging that “success in … management … does not rest on total cure but upon aggregate improvements”, services required entailed more than what physicians and nurses could provide, and a variety of therapies may be required. This led to the concept of therapeutic teams able to treat the patient as an “individual with diseases and disabilities rather than as a number of diseases and/or disabilities.” OT roles on these teams included teaching the patient to adapt to disability, helping them obtain and maintain as much personal independence as possible, and providing diversion/ occupation.
- Warren MW. Old People in Winter. The Medical Press and Circular. 1959, 242:420-24.
  - This examined the interplay between age and the seasons. In summer when older persons are out, “much can be learned about their medical conditions by watching them as they move about in the community.” During winter they are rarely seen. While most are well, medical conditions and infirmities are “more irksome during the inclemency of winter” with a higher likelihood of respiratory infections, aggravation of chronic medical conditions, falls, and “strained” social conditions (i.e., pinched finances, lack of help, isolation, inadequate housing).
- [Partial reference] Delore P, Warren MW, Riniker H. Rationalisation dans l’hospitalisation des malades de longue durée et des gens ages. Presse Med. (Supplément), 25 juillet 1959, n^o^ 36.
- Warren M. Mental Confusion in Elderly Persons. Geriatrics. 1959, 14(4):207-18.
  - While the belief that “some degree of mental failure in old age” was both inevitable and untreatable was “inadmissible”, Warren discussed four types of age-associated mental changes (i.e., senescence, reversible or temporary changes caused by physical conditions, irreversible or permanent changes with physical deterioration characteristic of senility, changes due to environmental conditions). (Note: she mentioned mental deficiency and mental illness as well, but these conditions were not dealt with further in this paper.) In assessing an older person for cognitive changes, knowledge of the “character of the old person himself in previous years” and the performance of someone with a similar background and age are needed. Warren used the term “senescence” for normal age-related changes and “senility” for pathological ones. Senescent changes included short-term memory loss, limited concentration, diminished mental reserve, and difficulty in adapting. Its severity ranged from very mild to a state close to senility that could be “accentuated by failing special senses and by an unsympathetic or unsuitable environment.” Reversible, temporary changes were what we now refer to as delirium. They were often related to dehydration, toxemia from infections or diabetes, anemia, uremia, cerebrovascular disease and “post-traumatic or postanesthetic shock.” Senile changes were greater than those seen with senescence and include more impairment or even complete loss of memory, disorientation to person and place, confusion, and development of “antisocial habits in some instances” (i.e., uncooperative, noisy, aggressive, doubly incontinent, “dirty in habits”). Causes would include “uncomplicated degeneration” without other diseases or “cerebral atheroma co-existing with other conditions.” Environmental factors associated with mental changes were retirement, isolation, and living “unhappily with members of his family, or as a lodger.” Warren noted how much was unknown and the need for research.
- Warren MW. J.B. Cook, M.D., D.P.H. Brit Med J. 1959, 1:654.
  - This was a contribution to the obituary of Dr. Cook, the former (1913-1945) medical superintendent of the WMH.
- Warren MW. Work Ability of the Aged. Brit Med J. 1959, 1(5113):33-34. (Book review)
- Warren M. The evolution of geriatric medicine. Geront Clin. 1960, 2:1-7.
  - At the beginning of this piece, Warren stated that geriatric medicine “must remain an integral part of general medicine if it is to fulfill its proper function.” Practitioners “do not set up to prolong life, but to make it easier and to treat patients as individuals rather than diseases.” Before its development, older patients when hospitalized were admitted to a medical ward under a general physician. If slow to respond, barriers to discharge were encountered, a bed was needed for a more deserving patient (i.e., younger, more acutely ill), or their “medical condition was not interesting to the physician” they would be transferred to a chronic sick institution for “permanent custodial care” even though many were still able to respond to active treatment. This arrangement was “for the patient medically inadequate, for medical staff frustrating, for the nurses uninteresting and for the country an intolerable economic burden.” Warren referred to her 1935 WMH survey where older patients were found “undiagnosed and medically untreated.” It “showed, without question, what was needed.” This was a full medical and social assessment, a detailed care plan based on this, and implementation of this plan by a positive therapeutic team consisting of physicians, nurses, ancillary staff, and social workers. With this approach, many would be able to return home under the medical care of their general practitioner (with outpatient supervision by the geriatric physician if needed). A “modern geriatric unit” consisted of an “out-patient and in-patient service with acute, long-stay, and convalescent sections … accepting patients direct from home” as well as from medical and surgical in-patient units. The general practitioners of older patients “recognize in the geriatric physician one who is particularly interested in the problems of the old and one who will ‘see his patients through.’” Education and research were other roles for these units. Warren acknowledged her “many medical colleagues”, singling out Drs. J Kaminski and M.M. Pam for special recognition.
- Warren M. Drugs in the Treatment of Old People. Brit Med J. 1960, 1(5189):1876-79.
  - While positive changes in pharmacotherapy had taken place, problems had arisen from the “spate of new drugs (often expensively and attractively advertised) which flood the market, and which the average doctor has too little time to study in detail.” The proper use of drugs starts with an accurate diagnosis. The paper summarized indications for certain drug classes (analgesics broadly defined, sedatives, tranquillizers) and suggested pharmacotherapy for common conditions (cardiac issues, infections, diabetes, Parkinson’s disease, blood diseases, ulcerated legs, constipation, anorexia). As general guidance Warren recommended “simple remedies, other than drugs, should always be given a trial first”, using small dosages, being aware of the risks of tolerance and abuse with analgesics and sedatives, and taking a “streamlined approach” (fewest drugs possible taken in the simplest way). D.G. Wood, Chief Pharmacist, WMH, was thanked for “reading through this paper.”
- Warren MW. Rehabilitation of the Elderly Patient. Rheumatology (formally Annals of Physical Medicine). 1960 (Feb), 5:170-81.
  - “It is necessary that his [the older patient’s] medical advisor should know something of the conditions likely to occur in a sick elderly person; that he should be able to prevent deterioration as often and as effectively as possible; and that he should know what active methods of approach and treatment to apply.” Rehabilitation in addition to knowledge and skills required “an attitude of mind to disabilities.” While additional invalidism could be prevented by “simple measures”, a rehabilitation program could be “successful only when undertaken by a therapeutic team” whose members were competent and understood their “own place in the team and the way in which he must consult with and advise others working for the patient.” Warren discussed the need for a “personal approach”, continuity of care, considering co-morbidities and what the patient will have to do post-discharge in developing rehabilitation plans, the utility of a pre-discharge home visit, having the patient even if incurable to do as much as possible for themselves for their morale (she would “insist” on this), and the use of “suitable gadgets” to achieve maximum possible independence. She thanked her colleagues with special gratitude extended to Dr. J. Kaminski.
- Warren M. Rehabilitation of Elderly Women. In: The Old Woman – Reports presented at the Extraordinary General Assembly of the Medical Women’s International Association (Baden-Baden, Germany, September 1960), pp. 41-51.
  - While driving to present this paper, Warren died in a single motor vehicle crash. Her paper was read by Miss Gertrude Herzfeld, the first female pediatric surgeon in Scotland. Much of it consisted of extracts from her 1958 *First Pan-Pacific Rehabilitation Conference* address and 1960 *Rheumatology* paper with nothing that dealt specifically with older women. In her writings Warren was not an advocate for the particular concerns of women. Sex/ gender differences were rarely noted. Though an active member of the Medical Women’s Federation (at the time of her death she was the newly elected President of the London Association of this organization; reference - AEBH. Marjory Winsome Warren. The Lancet. 1960, 2:656), her sister Enid was quoted as saying Warren felt “it was both unnecessary and unseemly to segregate women physicians in such gatherings” (Matthews DA. Dr. Marjory Warren and the Origin of British Geriatrics. J Am Geriatr Soc. 1984, 32(4):253-58).
    - Warren M. Rehabilitation of Elderly Women. Philippine Medical World. 1960, 21(5): 187. This summary of the above conference contribution was reportedly “by” Warren, but it is doubtful that she authored the paper.
- Warren M. Prescribing for Old People. In: Drugs in the Treatment of Disease – Specially Commissioned Articles from the British Medical Journal. British Medical Association (London), 1961, pp. 465-74. (This was to have been a revised version of the 1960 *Brit Med J* article on drug therapy, but because of her death no changes were made.)
